# Feasibility Study on the Generation of Nanoporous Metal Structures by Means of Selective Alloy Depletion in Halogen-Rich Atmospheres

**DOI:** 10.3390/ma17020498

**Published:** 2024-01-20

**Authors:** Jörg Weise, Birgit Uhrlaub, Dirk Lehmhus, Joachim Baumeister, Kerstin Hantzsche, Karsten Thiel

**Affiliations:** 1 Fraunhofer Institute for Manufacturing Technology and Advanced Materials IFAM, Wiener Strasse 12, D-28359 Bremen, Germany; birgit.uhrlaub@ifam.fraunhofer.de (B.U.); dirk.lehmhus@ifam.fraunhofer.de (D.L.); joachim.baumeister@ifam.fraunhofer.de (J.B.); karsten.thiel@ifam.fraunhofer.de (K.T.); 2 Leibniz Institute for Materials Engineering—IWT, Badgasteiner Str. 3, D-28359 Bremen, Germany; hantzsche@iwt-bremen.de

**Keywords:** porous materials, nanoporous metals, alloy depletion, dealloying, halogen

## Abstract

A new approach to produce nanoporous metals has been investigated, which is based on the dealloying of bi- or multi-component alloys. Depletion and pore formation of the alloy substrate are obtained by the transport of certain alloy components at high temperatures via volatile halogen compounds. These halogen compounds are transferred to materials acting as sinks based on their higher affinity to the respective components, and chemically bound there. Transfer via volatile halogen compounds is known from the pack cementation coating process and from high-temperature corrosion in certain industrial atmospheres. The approach was tested on different precursor alloys: Ti-43.5Al-4Nb-1Mo-0.1B (TNM-B1), TiNb42, and AlCu. Both dealloying effects and micro-scale pore formation were observed. The detailed size of the porous structures is in the range of 50 nm for both TNM-B1 and TiNB42 and 500 nm for AlCu.

## 1. Introduction

Nanoporous (NP) metals have raised considerable industrial and scientific interest because of functional properties such as high internal surface area, improved catalytic activity, and sensing capabilities [1,2]. The most famous example of NP metals is Raney nickel [3], which is used as a catalyst in organic chemistry. Today, various NP metals (such as Au, Cu, Fe, Co, and Nb) and application fields (energy conversion, storage, and sensing) are being investigated [1,4].

A large variety of processes for the preparation of NP metals has been developed, e.g., free corrosion and electrolytic dealloying in aqueous solutions, air-free electrolytic dealloying in organic electrolytes, vacuum thermal dealloying, and reduction-induced decomposition or liquid metal dealloying [5,6,7]. The most common approach is the dealloying in aqueous solutions, i.e., the selective dissolution of one or more components from an alloy [5]. Under certain kinetic conditions—if the dissolution rate is fast enough relative to the surface diffusion rate of the undissolved component—the formation of nano-porosity in the substrate, which is enriched with the more noble alloy components, can arise [8]. Examples of structures produced by means of this approach are NP Au, Ag, Pt, or Cu structures with the use of precursor alloys such as Au-Ag, Au-Cu, Ag-Al, Pt–Cu, or Cu–Mn [5,9].

However, when NP structures made from less noble metals are striven for, dealloying in aqueous solutions is often no longer a viable option. Therefore, for metals such as Ti, Fe, Zr, or Nb, the so-called liquid metal dealloying (LMD) process was proposed [10,11,12]. In this process, the substrate to be dealloyed is immersed into a liquid metal melt (e.g., Mg). Several components of the substrate diffuse and dissolve into the metal melt, whereas the remaining component or components are immiscible in the melt and rearrange into a nanoporous scaffold. Although this technique allows the processing of metal alloys which cannot be approached by other methods, LMD has several drawbacks. Magnesium melts, which are often used, are very reactive, thus special furnace equipment has to be employed, and handling is difficult. Furthermore, resource use is significant because the compound uptake capability of the molten metal sink is limited by compound solubility. After the process, the metal melt has to be removed from the porous structure, e.g., by etching in HNO_3_ [13]. Generally, LMD relies on substrate geometries such as thin ribbons. The processing of powders, which would be very interesting for many applications, is very difficult. 

**Figure 1:**
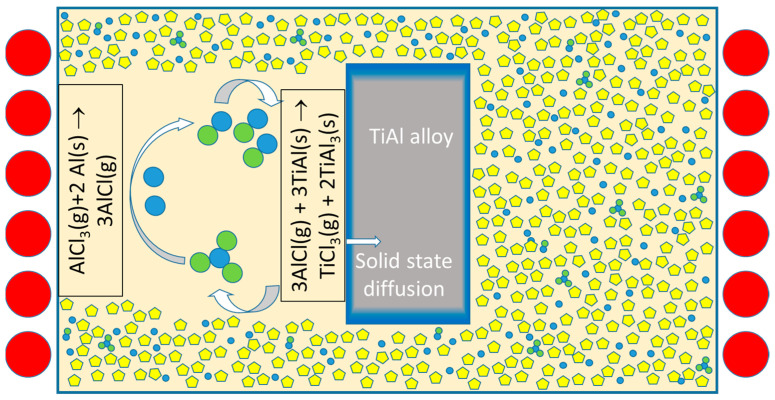
Schematic setup and transport principle of the pack cementation process (example of alumination of the TiAl alloy; blue: aluminium, green: chlorine, yellow: inert filler).

As already outlined, the need for direct contact between the melt and the precursor substrate is a characteristic feature of LMD, but also the cause of several of the above-mentioned problems. The main question is whether the transport of alloy components between the precursor alloy substrate and the “sink” can be maintained without direct contact, avoiding the problems associated with LMD. Indeed, such methods exist, with pack cementation being the most prominent example. In this process, alloy components are transferred from a “powder pack” to a substrate at a high temperature via volatile metal compounds to produce diffusion coating zones at the surface of the substrate [14,15]. Usually, a substrate made from steel or superalloys is embedded into a mixture of inert powder (e.g., Al_2_O_3_) and aluminum powder, and about 1–2 wt% of an activator is added to this pack (e.g., aluminum chloride, ammonium chloride, or other ammonium halides). The pack is then heat-treated at 800–1000 °C in an Ar or H_2_ atmosphere. This causes the formation of volatile aluminum halides, which diffuse through the pack, react with the substrate, and deposit aluminum which diffuses into the substrate surface, see Figure 1. Pack cementation is a special variant of a chemical vapor deposition (CVD) technique and is often used to generate surfaces enriched with Al, Cr, or Si in order to improve the oxidation resistance of ferrous and other alloys. In this process, thanks to the volatile compounds, no direct contact between the aluminum source and the substrate is needed for the transfer of the aluminum component. 

Based on this, one could envisage the reverse process, i.e., the transport of components from a precursor alloy substrate to a sink in which these components are accumulated. This way, the precursor alloy can be depleted of certain components and nanoporosity can be generated in a way similar to LMD. In the deposition of chromium-aluminide coatings by means of pack cementation, using Cr-Al master alloy particles in the pack, aluminum depletion was observed for the Cr-Al particles [16]. However, to the authors’ knowledge, so far this effect has not been exploited directly for the production of NP metals.

Several requirements must be met for such a reverse pack cementation process and the generation of nanoporous structures:

- The precursor substrate must consist of two groups of components: the components remaining in the structure after the dealloying and the components which shall be removed from and depleted in the precursor. For the latter, it is indispensable that they form volatile halogen compounds. In contrast, for the first group, it is advantageous if no such volatile compounds are formed. Generally, the affinity of the first group to the halogen activator should be low to prevent bonding of the activator which would then be lacking for the transport process.
- None of the components should form poisonous or other dangerous compounds with the halogen activator.
- In the pack, a “sink” material (block, melt, or powder) must be available for the uptake of the components which shall be removed from the precursor. Therefore, the bonding or mixing enthalpies of the components in the precursor and the sink must be appropriate.
- It is advantageous (but not essential) that the sink material does not form volatile halogen compounds. If the sink forms such compounds, both the transport of components from the precursor to the sink and the transport of the sink material to the precursor are possible, making subsequent cleaning operations for the dealloyed precursor necessary.
- The quantity of the sink material must be sufficient for the uptake of the depleted components.
- Finally, as for other dealloying techniques, the rates of component depletion and (surface) diffusion in the precursor material must be appropriate in order to allow the formation of nanoporous structures. This has consequences for the choice of appropriate treatment conditions (especially the process temperature), which must ensure sufficient formation and transport rates of volatile compounds in combination with limited solid-state diffusion in the precursor substrate.

Similar to LMD, metal and especially magnesium melts or powders could be used as a sink in a reverse pack cementation process. In this case, a high solubility, resp. appropriate solution enthalpies, of the components which shall be removed from the precursor is needed in the metal. Contrary to this, the other components in the precursor should exhibit a very low or no solubility in magnesium. However, a disadvantage of magnesium is that it also forms volatile halogen compounds [17]; therefore, the transport of magnesium to the precursor substrate and the need for subsequent cleaning procedures such as in LMD-processing must be expected. 

Temperature gradients can also be used as thermodynamic driving forces for the transport and deposition of alloying elements. This approach was used in the Gross resp. sub-halide process, which was developed by the company ALCAN to distill technically pure aluminum from “crude” aluminum alloys (containing very high levels of Si and Fe) [18,19]. In this process, the different equilibrium conditions of the disproportionation reaction

2Al(l) + AlCl_3_(g) = 3AlCl(g) (1)

in two connected vessels with different temperature levels (converter and condenser) were exploited. However, the process temperatures in the converter (ca. 1200 °C), in which the aluminum depletion occurs, are very high. Even if the depletion leads to the formation of a porous structure, rapid coarsening effects have to be expected. 

A third approach is to bind the components chemically, e.g., in the form of oxides. Oxygen sources could ensure oxygen content levels in the chamber’s atmosphere, such as humidity or oxides which can easily be reduced when in contact with the volatile halogen compounds. For this approach, close similarities to mechanisms discussed for high-temperature corrosion processes in industrial atmospheres (metal–chloride–oxide cycle) exist [20,21]. 

Furthermore, inorganic non-metallic compounds can act as potential sinks for certain alloy components, taking advantage of the so-called solid-state ion exchange techniques for zeolites. In these techniques, metal halides are frequently applied [22]. Anderson et al. showed that aluminum is isomorphously substituted for silicon in the zeolitic framework and also enters 6-coordinated (octahedral) intrazeolitic positions when highly siliceous zeolite is treated with AlC1_3_ vapor at elevated temperatures [23]. In addition, Lang et al. described the AlCl_3_ modification of siliceous microporous and mesoporous catalysts [24]. In the case of substitution of Al atoms with other elements in the zeolite framework, one must consider, of course, the generation of respective volatile halides, which can in turn react with the alloy substrate to be dealloyed. Another example of transformation processes based on transport via volatile halogen compounds is the synthesis of aluminum carbide-derived carbon, as reported by Moran et al. [25]. Of course, in all these cases, the respective thermodynamic driving forces must be taken into account. 

In our own investigations, we experimentally tested the hypothesis of whether reverse pack cementation resp. oxidation in halogen-rich atmospheres can purposefully be used for the dealloying of metal substrates and the generation of porous structures with sub-micron pore sizes. 

## 2. Materials and Methods

**Figure 2:**
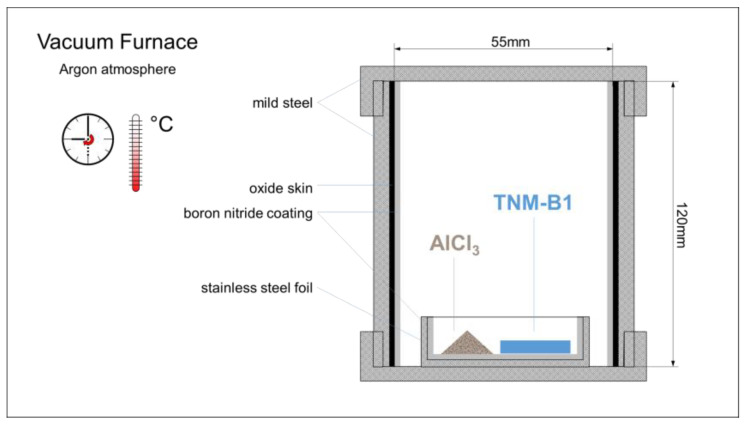
Schematic drawing of the experimental setup for the dealloying of a TNM-B1 alloy block.

For the first experiment, bulk TNM-B1-alloy (Ti-43.5Al-4Nb-1Mo-0.1B, by courtesy of University of Technology Cottbus-Senftenberg, Cottbus, Germany) was used as a precursor alloy. Small cylinders (d = 10 mm) were sectioned into smaller plates. One precursor alloy plate (1 g) was put into a stainless-steel cap, which was coated with BN. Then, 0.13 g AlCl_3_ (Merck, Darmstadt, Germany) was added as an activator. The mass ratio of precursor alloy and activator was increased in comparison to values typical of the pack cementation process in order to intensify gaseous transport in contrast to solid-state diffusion within the precursor [14]. The foil cap was placed into a mild steel pipe (d_i_ = 55 mm, h = 120 mm, V ≈ 285 cm^3^, see schematic Figure 2). The mild steel featured a thin internal oxidation scale, obtained by earlier heat treatment in air (used as an internal oxygen source in the experiment). In order to prevent overly intense reactions between the steel wall and the activator, the internal pipe surfaces were sprayed lightly with BN and dried. The pipe was closed at both ends with threaded caps and sealed with ceramic wash (this was a safety measure in order to avoid the emission of the activator from the pipe and unwanted reactions in the furnace). The closed and sealed pipe was put into a vacuum furnace (Thermal Technology, Bayreuth, Germany) and heat treated in an argon atmosphere for 4 h at 800 °C. This temperature is at the lower margin of the temperature range of industrial pack cementation processes for high melting alloys, again in order to limit solid state diffusion within the precursor [14]. A similar experiment was conducted without the addition of the AlCl_3_ activator as a reference.

After the heat treatment, the different TNM-B1 specimens were analyzed by scanning electron microscopy and a focused ion beam in a FEI Helios 600 Dual-Beam machine (ThermoFisher Scientific, Eindhoven, The Netherlands). Elemental analysis was performed by EDS using an Oxford X-Max80-EDS detector (Oxford Instruments Nanoanalysis, Wiesbaden, Germany) with an energy resolution of 129 eV @ Mn-Kα. Furthermore, as a reference, an untreated TNM-B1 specimen was analyzed metallographically.

As described in the introduction, in contrast to processes like LM, dealloying via gas-phase transport could facilitate the treatment of powders. Therefore, an additional test with TNM-B1 powder was performed. TNM-B1 (Ti-43.5Al-4Nb-1Mo-0.1B, fraction 90–180 µm) powder was produced and provided by Helmholtz-Zentrum Hereon, Geesthacht, Germany. A total of 0.5 g of precursor powder was mixed with 0.045 g of the activator AlCl_3_ and processed analogously to the bulk specimen. The treatment time was extended to 10 h in consideration of the much larger surface to be dealloyed. Similar to the bulk specimen, after the treatment the powder was covered with an oxide scale (see below). In order to peel the oxide scale off and expose the dealloyed metal below, the powder was very softly ground between two stainless steel cylinders. Again, structure evaluation was performed using SEM.

To demonstrate the applicability of the dealloying approach to other alloys, a test with Ti-42 at%Nb powder (AMtrinsic^®^ Spherical Ti-42Nb, TANIOBIS GmbH, Goslar, Germany) was performed: 0.056 g AlCl_3_ activator was added to 0.55 g precursor powder. Treatment time and temperature were chosen based on the example of TNM-B1 (4 h and 800 °C) as both alloys have comparatively high melting temperatures. 

In all experiments mentioned above, reducible oxides in the container were used as oxygen sources. In one additional experiment, it was tested whether humidity could be employed as an alternative oxygen source. For this experiment, an Al-Cu precursor was used as this is a well-known system for the production of NP metals. The Al-Cu precursor alloy was prepared by mixing 8.1 g Al shot (99.999%, Alfa Aesar/ ThermoFisher Scientific, Schwerte, Germany) and 18.9 g Cu shot (99.9+% Alfa Aesar/ThermoFisher Scientific, Schwerte, Germany) in an alumina crucible, followed by melting in an induction furnace (Bego Fornax, Bego GmbH & Co. KG, Bremen, Germany). The melt was manually stirred with a silica rod. The composition of 30 wt% Al and 70 wt% Cu was chosen to obtain a structure dominated by the η_2_-(Al_48_Cu_52_) phase. After solidification, the alloy block was broken and parts were ground manually into small powder particles. Immediately after grinding, 0.6 g of the powder was mixed with 0.05 g of AlCl_3_ activator and poured into an alumina crucible (d = 5 cm), filled almost completely with Y-zeolite granules (Na-Y-BFK, CWK Chemiewerk Bad Köstritz GmbH, Bad Köstritz, Germany, appr. 30 g, d ca. 2 mm). The residual water content of the zeolite was determined (by heating the as-received zeolite to 250 °C and measuring the mass change) to be 2 wt%. The crucible was lightly tapped in order to obtain a good distribution of the fine powder in the bulk of granules. The crucible was closed with stainless steel foil and 4 layers of additional Al foil. The closed crucible was transferred into a vacuum furnace. The thermal cycle was as follows: evacuation, filling with argon, heating at 10 K/min to 250 °C, holding for 65 h, and cooling to RT. The lower process temperature was chosen because of the relatively low melting temperature of the Al-Cu alloy and in accordance with the work of Christoglou et al., who investigated pack cementation coatings on magnesium [15].

Afterwards, the powder mixture, which had changed its appearance from metallic gray to copper-colored, and the zeolite granules were separated, and the precursor powder was analyzed by means of SEM using a Dual Beam Helios G4 PFIB CXe (ThermoFisher, Brno, Czech Republic) and EDS-System Octane Elite Plus, EDAX-Ametek, Pleasanton, CA, USA.

## 3. Results

### 3.1. TNM-B1 Alloy Block and Powder

**Figure 3:**
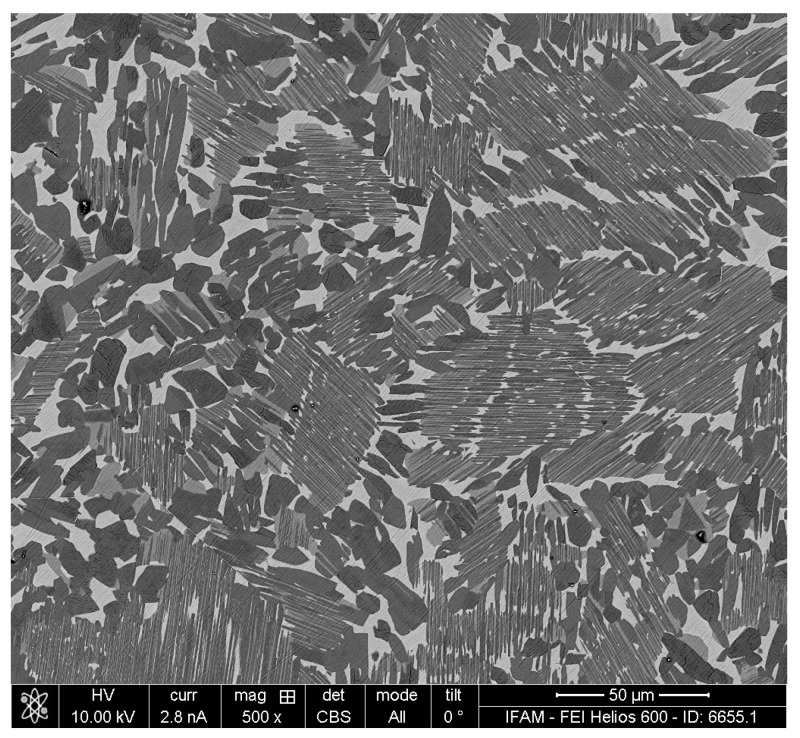
Polished surface of a TNM-B1 specimen, original state, SEM image—dark: γ-TiAl, grey: α2-Ti_3_Al, and bright: β- TiAl.

The original TNM-B1 alloy cylinder with the nominal composition Ti-43.5Al-4Nb-1.0Mo-0.1B (in at%) exhibits a three-phase microstructure (Figure 3), consisting of the γ-TiAl, α2-Ti_3_Al, and β-TiAl phases, compared with Figure 1 in [26,27]. 

**Figure 4:**
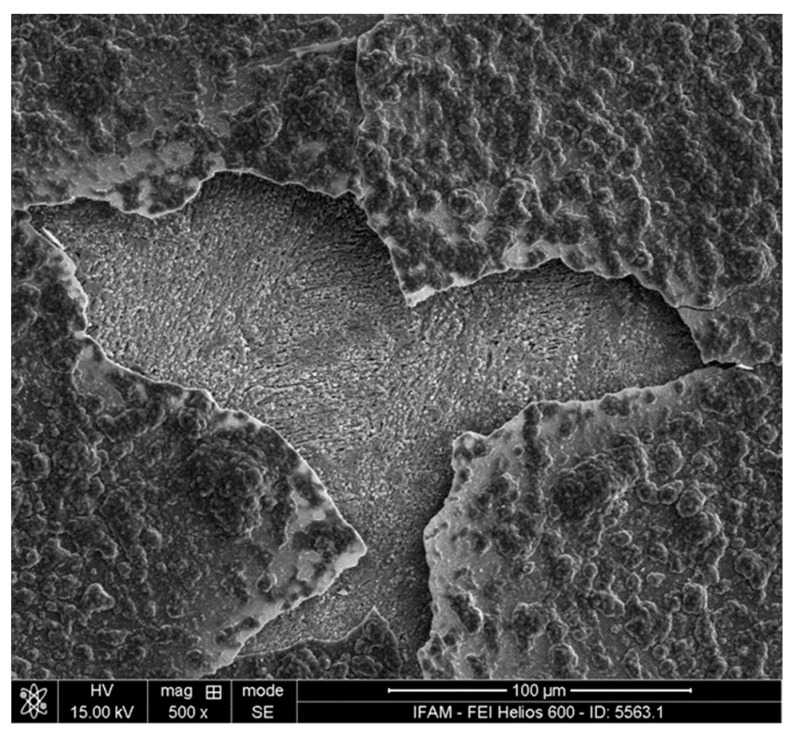
Surface of a TNM-B1 specimen heat-treated with a halogen activator, featuring a partly spalled oxide scale.

**Figure 5:**
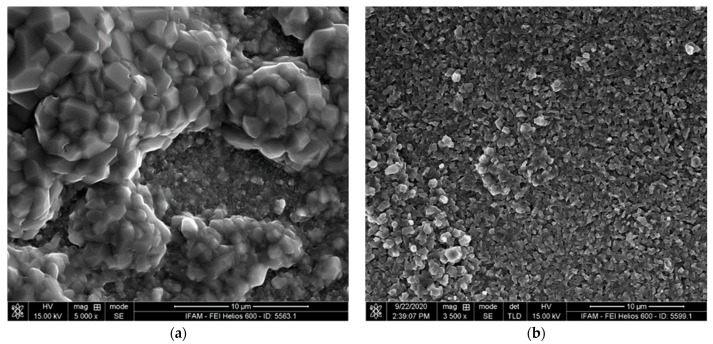
SEM image of the oxide scale’s surface: (**a**) TNMB-1 heat-treated with an activator; (**b**) TNMB-1 heat-treated without an activator.

**Figure 6:**
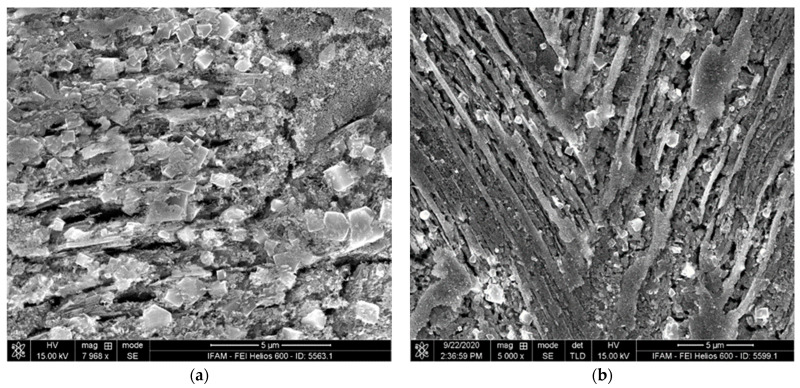

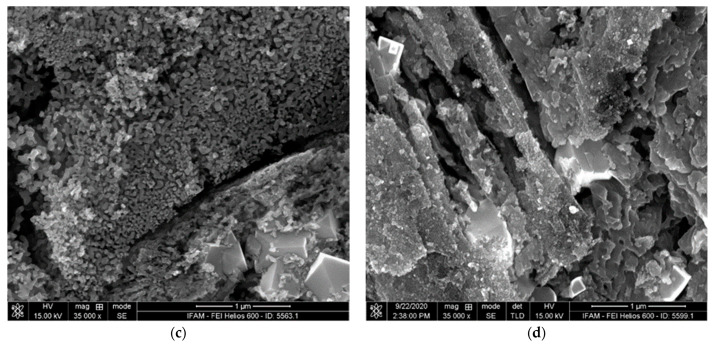
Substrate surface below the oxide scale: (**a**,**c**) TNMB-1 heat-treated with an activator at different magnifications; (**b**,**d**) TNMB-1 heat-treated without an activator at different magnifications.

The specimens heat-treated with and without a halogen activator showed very similar macroscopic appearances: a rough surface with a partly spalled oxide scale (see the example in Figure 4). However, on a microscopic scale, both the oxide scale and the underlying substrate surface differ considerably (Figure 5 and Figure 6). The oxide scale of the activator-treated specimen exhibits bulbous structures on top of a finer crystalline layer. The reference specimen only shows the latter. The substrate surface below the scale of both specimens is very rough. Three different, typical structural elements can be distinguished:

- small facetted crystals with smooth surfaces
- plate-like structures with relatively smooth lateral faces
- rough (dark grey) areas

As can be seen in Figure 6, the latter areas exhibit porous structures in the case of the activator-treated TNM-B1 (Figure 6a,c), whereas the corresponding regions of the reference specimen are characterized solely by very rough surfaces (Figure 6b,d). 

**Figure 7:**
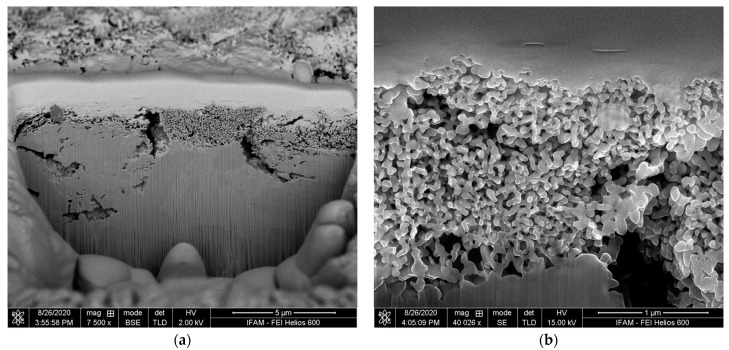
FIB section through the sample: (**a**) overview of sectioned area; (**b**) detailed view at a higher magnification, showing the porous structure created by the treatment.

To evaluate the depth of the porous layer of the activator-treated specimen, a FIB section orthogonal to the surface was prepared (see Figure 7). The layer has a coralline appearance and its maximum thickness is approximately 1.5 µm. The observed variation in thickness can be attributed to the multi-phase character of the precursor alloy. Typical strut diameters of the porous structure are about 50 nm.

**Figure 8:**
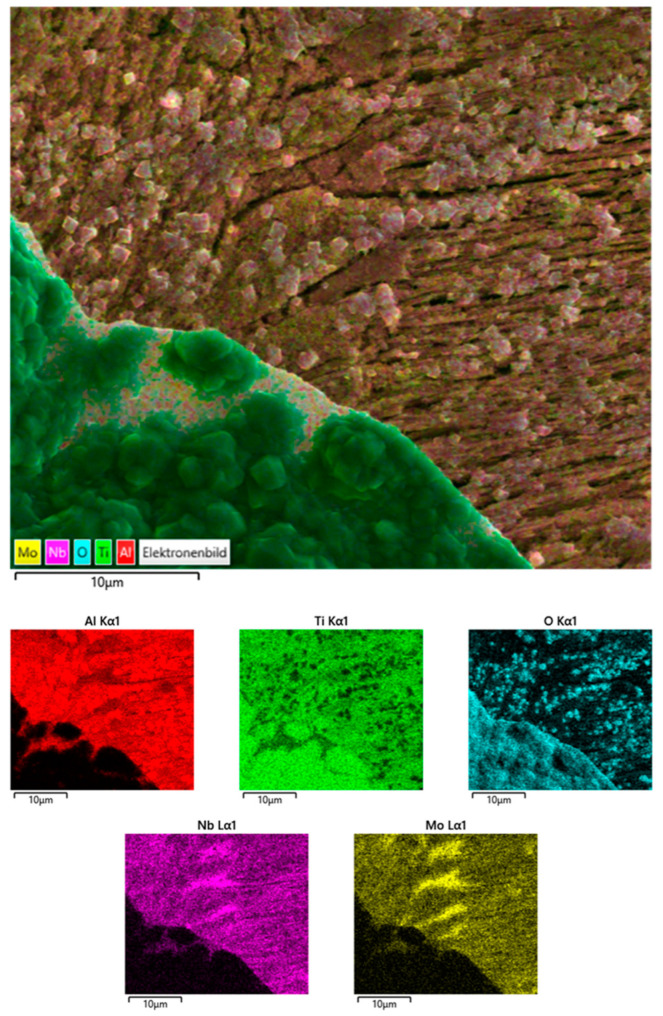
SEM-based EDS area mapping of the surface of the activator-treated TNM-B1 sample with the oxide scale in the lower left part of the image.

Figure 8 shows the results of the EDS mapping of the surface of the activator-treated specimen with a partly spalled oxide scale. Clearly, Ti and O dominate in the outer nodular zone of the oxide scale. The fine-crystalline oxide layer below the nodules seems to consist of alumina. Furthermore, there is a strong indication that the small smooth crystallites on the surface below the scale are also alumina. No traces of Nb and Mo are detected in the nodular scale. EDS measurements on the FIB section (Figure 7) showed an increase of the Nb and Mo contents compared to the original precursor content of 4 at% to 14 at% and 1 to 7 at%, respectively, whereas the aluminum content dropped from 43.5 to 33 at%. The Ti content changed slightly from 51 to 46 at% in comparison to the original material. 

**Figure 9:**
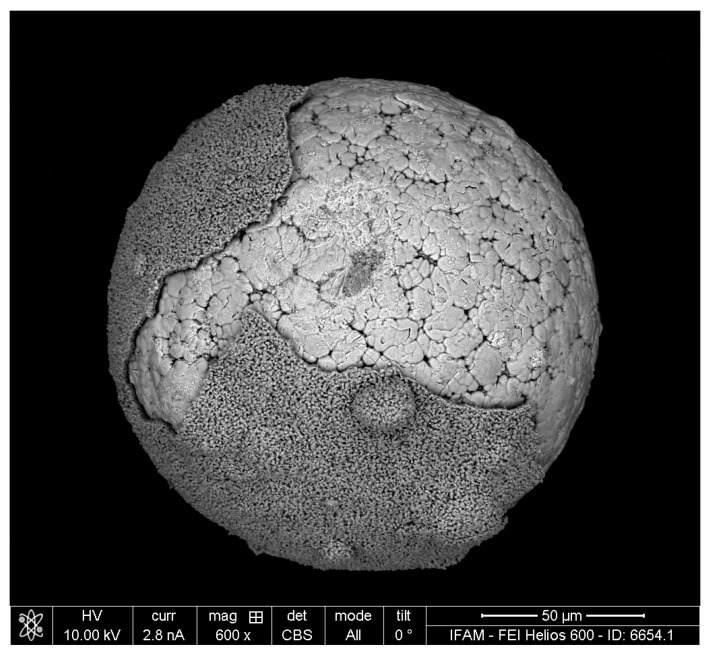
SEM image of the TNM-B1 particle with a partly removed oxidation scale.

**Figure 10:**
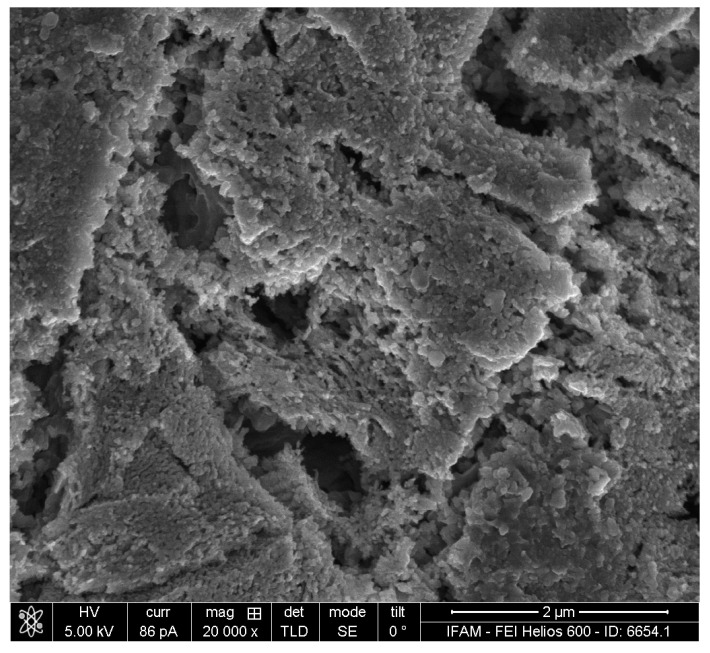
SEM image of the TNM-B1 particle after the dealloying treatment, showing the porous surface structure.

The results obtained for the TNM-B1 powder closely followed the observations made on the block material. In Figure 9, an activator-treated particle is shown after soft grinding. Obviously, the scale detaches easily from the underlying metal surface. The macroscopic appearance of scale and metal is quite similar to what is seen on the activator-treated alloy block (compared to Figure 4). One difference is the more dendritic surface topography of the powder particle’s metal surface, which can be attributed to the higher solidification rate during the production of the powder particle. Higher-resolution SEM images reveal a porous structure of the metal, with pore sizes in the range of 50–100 nm (Figure 10). EDS mapping and area measurements showed that the scale is oxidic. Excluding oxygen, the main metal component of the scale is Ti with 88 at%. The Al content is reduced to only 6.5 at%. In addition, an Fe content of 4.3 at% is found. Furthermore, small traces of Si, V, and Cl were also found. In contrast, neither Mo nor Nb were found in the scale. In the porous structure, the EDS results are (in at%): Ti: 53, Al: 36, Fe: 1.6, Nb: 6.6, and Mo: 1.7, as well as small traces of Si and Cl (in comparison, EDS results for the original, untreated powder revealed (in at%): Ti: 55, Al: 40, Nb: 4, and Mo: 1). 

### 3.2. Ti-Nb Alloy

**Figure 11:**
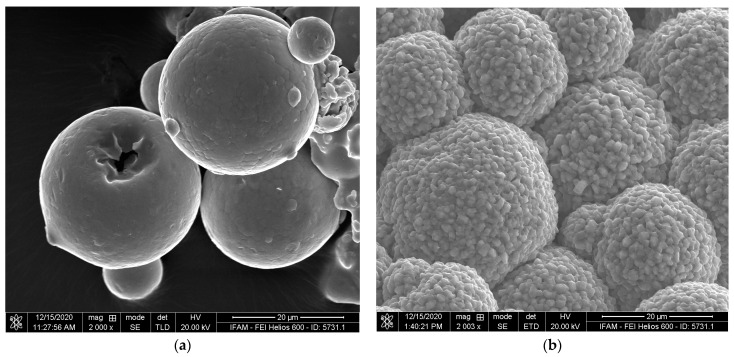
SEM image of Ti-42 at%Nb powder particles (**a**) before and (**b**) after activator treatment.

**Figure 12:**
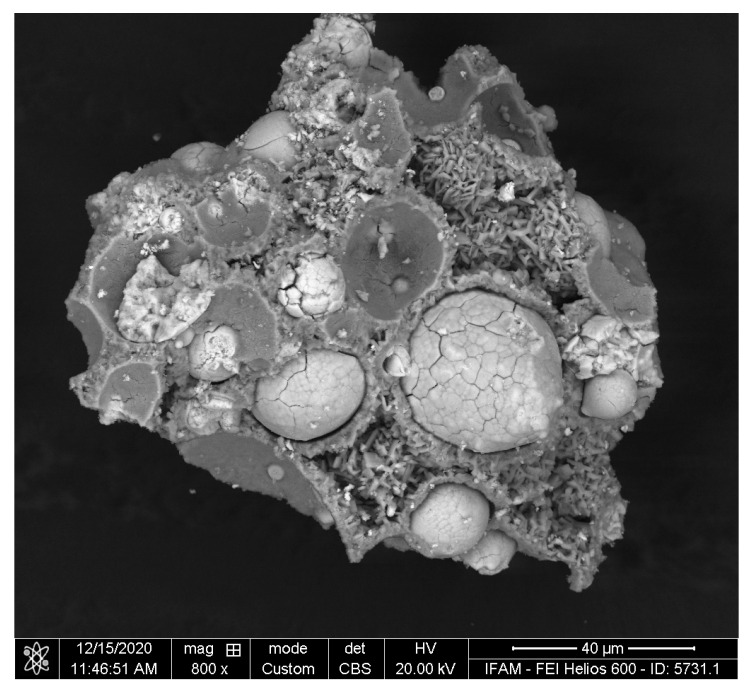
SEM image of Ti-42 at%Nb powder particles after activator treatment and soft grinding.

The macroscopic appearances of Ti-42 at%Nb powder particles before and after activator treatment are compared in Figure 11. A thick layer with irregularly formed crystallites, connecting groups of powder particles, can be observed in the latter case. The layer can easily be separated from the core of the particles by soft grinding (Figure 12).

**Figure 13:**
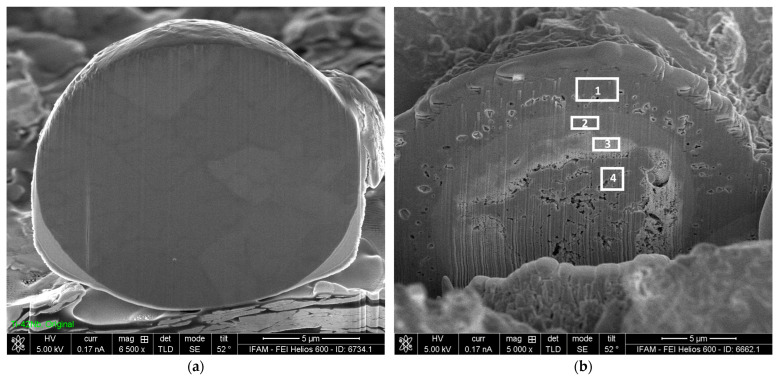
FIB section through Ti-42 at%Nb powder particles. (**a**) non-treated, (**b**) activator-treated with indication of EDS measurement areas as discussed in the main text.

FIB sections of an original and an activator-treated particle are shown in Figure 13. The SEM figure of the untreated particle indicates a single-phase dendrite structure with slight segregations between the dendrite core and border. The original powder does not reveal any porosity. In comparison, the activator-treated particle exhibits a complex layered structure. Altogether, four layers were identified:

1. an outer scale with coarse pores close to the interface of the second layer
2. an intermediate dense layer
3. a second porous layer
4. a core with a coarse internal pore structure

The separation of layers during grinding seems to occur at the interface between the second porous layer and the core. 

**Figure 14:**
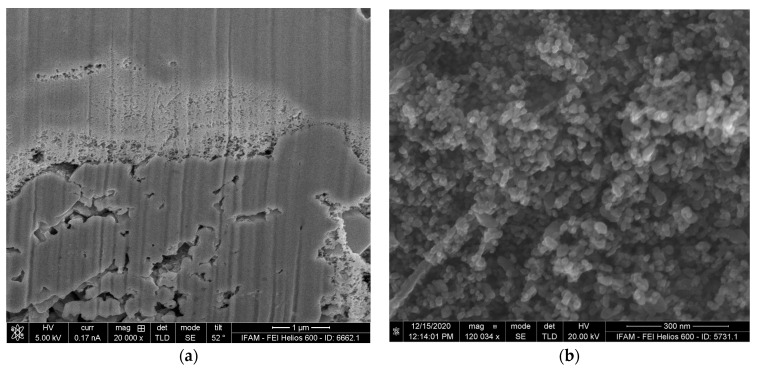
High-resolution SEM image of the porous intermediate layer, (**a**) FIB section, (**b**) broken sample. Note the difference in magnification.

In Table 1, the results of EDS area measurements are shown. The outer layer is rich in Ti and O, suggesting TiO_2_ as the main phase. The other layers exhibit lower O contents. In any case, oxygen contents determined by EDS have to be assessed very critically, especially for such small measurement areas. Both the dense and the porous intermediate layers are enriched in Nb, especially the porous layer as the ratio of Nb to Ti is high at 1:56 after the dealloying treatment. In Figure 14, the structure of the porous layer is presented, indicating typical detail sizes of 20–50 nm. The porous core exhibits a Nb:Ti ratio close to that of the original precursor alloy.

### 3.3. Cu-Al Alloy

EDS measurements of the untreated Al-Cu powder showed a ratio of Cu to Al atoms of 1.05, which correlates well with the intended precursor composition. After the dealloying treatment, in the stereomicroscope images, two kinds of particles could be distinguished: the majority were copper-colored with a rough surface. Other particles, especially larger ones, still had a shiny appearance. This indicates that, if any dealloying occurred, the reaction was not completed. 

**Figure 15:**
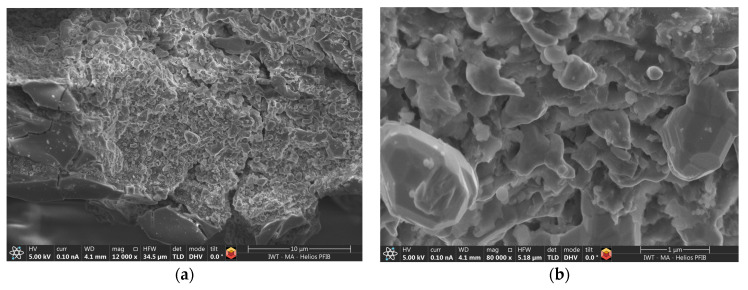
SEM image of the porous structure of the dealloyed AlCu specimen. (**a**) overview of smooth particle surface and exposed underlying porous layer, (**b**) detail image of underlying layer.

The SEM investigations showed that many treated particles appeared torn up and spalled. The outer surfaces appear smooth whereas the exposed internal areas are porous with a plate-like and rather irregular structure (Figure 15). The smooth outer surfaces (and the first underlying layer) had Cu-Al ratios close to 1 and a considerably increased oxygen content. 

The detailed size of the porous internal structure was approximately 0.5–1 µm. The EDS analysis at various points of the porous structure revealed that the Cu:Al ratio was shifted to values of 4.2–15.2. Both dealloying and structuring effects could, therefore, be verified. However, such a shift was not observed in all investigated areas, confirming the initial impression that the reaction was still incomplete. The rather coarse porous structure might indicate that the ratio of mass transfer via volatile compounds and solid-state diffusion is unfavorable in the case of Al-Cu, even at the rather low process temperatures maintained in the present experiments. However, no final proof of this assumption could be derived in the course of this study. 

## 4. Discussion

A survey of the experimental results for the different alloys and component sinks allows the conclusion that transport via volatile halogen compounds can indeed lead to dealloying effects and to the formation of porous structures on the sub-micron scale. Nevertheless, the examples shall be discussed individually.

### 4.1. TNM-B1 Alloy Block and Powder

The basic experimental setup for treating the TNM-B1 block and powder closely follows the pack cementation process. However, in the current experiment, there are special process conditions. Especially the amount of oxygen-affine metal powder, which is the focus of our investigation, the container volume and the available oxygen residues in the system differ largely from those of the PC process. Unsurprisingly, for both experiments with and without the AlCl_3_ activator, superficial oxidation of the TNM-B1 powder was observed. Therefore, it is worthwhile to compare the results of this work with observations and mechanisms discussed in the literature on high-temperature corrosion (e.g., [20,21]). 

The macroscopic and microscopic appearance of the activator-treated TNM-B1 alloy specimen bears some resemblance with the observations of Laska et al., even though the treatment conditions differed in several aspects (1000 1 h cycles of exposure at 850 °C in laboratory air) [28]. In both cases, the scale consisted of outer nodules of TiO_2_ with an underlying non-nodular zone, in which alumina seems to have formed preferentially. At various areas, the oxide scale spalled off. In addition, Laska et al. found Nb- and Mo-rich precipitates at the interface of scale and substrate, in analogy to the Nb and Mo enrichment observed for the activator-treated TNM-B1. In comparison, the TNM-B1 reference specimen differs more, as the nodular layer is missing in this case. It can be assumed that the main reason for this is the large difference in treatment time; 4 h versus 1000 h in the present work versus the work of Laska et al. The closer similarities in the case of the activator-treated TNM-B1 can be explained by a more severe oxidation attack caused by the action of the halogen. 

Depending on the specific conditions, halogens such as chlorine can act either by accelerating or retarding the oxidation rate of TiAl alloys. The specific interactions of chlorine-based gas-phase transport and deposition are discussed in detail in [20,21], which demonstrated that:

- chlorine is transported from the atmosphere through a diffusion boundary layer to the material surface;
- reactants diffuse through the oxide scale;
- chlorine reacts with metal or metal oxide;
- volatile metal chlorides are formed;
- volatile metal chlorides diffuse through the oxide scale in an outward direction;
- volatile metal chlorides react with oxygen from the environment along the outward-orientated transport path through the oxide scale with increasing oxygen partial pressure, forming chlorine and solid metal oxide;
- chlorine re-diffuses from this reaction to the metal-oxide phase boundary.

Depending on the available alloy components and their individual chemical behavior (formation of chlorides, oxides, and oxy-chlorides), the reaction temperature, and the atmosphere composition, this chloride–oxide cycle can lead to the formation of protective dense alumina layers, which efficiently prevent further oxidation [29,30]. This “halogen effect” is used purposefully in the so-called halogen-implantation of TiAl components. On the other hand, if this chloride–oxide cycle does not lead to the formation of protective oxide layers, an accelerated oxidation is the result. The latter seems to be the case in our experiment. 

According to [20], for Cl vapor pressures > 10^−5^ bar, considerable formation of volatile metal chlorides (and considerable transport of metal via the chlorides) can be expected. Only in a certain range of O and Cl partial pressures is the formation of TiCl_3_ suspended (see Figure 12 in [31]), whereas the formation of AlCl_3_ continues, leading to the formation of a dense, protective alumina layer. In comparison, for higher Cl partial pressures, both Ti- and Al-chlorides will form, leading to a non-dense scale. In the experimental setup for the TIM-B1 alloy, the added AlCl_3_ masses are equivalent to 0.045 mol and 0.13 mol, respectively. This translates to 7.5 cm^3^ or 21.8 cm^3^ when the AlCl_3_ has evaporated and, according to the ideal gas law, to partial pressures of 0.026 bar and 0.07 bar. For the high chlorine content in the system in the experiments presented, an acceleration of the oxidation rate can be expected. However, the overall situation is quite complex, as with Ti, Mo, Nb (substrate), and Fe (wall), several components, capable of forming volatile halides, are available. Specific reactions for the formation of halides such as AlCl, AlCl_2_, and TiCl_3_ can be found in [32,33,34]. According to the Gibbs free energies of reaction for the formation of metal chlorides per mole chlorine as given by [20], as well as Figure 3 and Figure 4, the formation of volatile Mo and Nb chlorides is much less favorable at the treatment temperature of 800 °C: AlCl_3_: −350 kJ/mol, TiCl: −320 kJ/mol, NbCl: −220 kJ/mol, and MoCl: −150 kJ/mol (approximate values). However, due to the high Cl content used in our experiment, the formation of Mo- and Nb-chlorides cannot be excluded completely (compared to [20], Figure 5, Figure 6 and Figure 9). 

The role of iron is of specific interest as it can act as an oxygen source in the system. Fe and its oxides can react with AlCl_3_ to form FeCl_3_ (or its dimer Fe_2_Cl_6_) [35,36]. In the case of the reaction with oxides, aluminum-oxychloride AlOCl is also formed according to [35]:

2 Fe_2_O_3_(s) + Al_2_Cl_6_(g) → 6 AlOCl(g) + 2 Fe_2_Cl_6_(g). (2)

Quasi-stability diagrams of iron, iron oxides, and iron chlorides show that under the present treatment conditions, the formation of FeCl_2_ according to this reaction is possible (see [20], Figure 15, or [37], Figure 2). The volatile oxychloride formed according to Reaction (1) allows the transport of oxygen from the container wall to the substrate and its reaction with Al and Ti, leading to the formation of an oxide scale (as a component sink) and an Al- and Ti-depleted substrate. An important factor for this is the differences in driving force for the oxidation, i.e., the Gibbs free energy for the formation of the respective oxides of the alloy’s components with the approximate values of Mo: −520 kJ/mol, Nb: −580 kJ/mol, Ti: −720 kJ/mol, and Al: −900 kJ/mol at the treatment temperature of 800 °C. 

Altogether, the above-mentioned data regarding chloride and oxide formation match the experimental observation of the formation of Al- and Ti-rich oxides at the surface of the treated TNM-B1 specimens as well as the superficial substrate zone depleted of Al and Ti. This was observed both for the reference and the activator-treated TNM-B1 specimen. Obviously, the oxide–chloride cycle can be active only in the latter case. Although the details are still not fully understood, it can be supposed that the higher oxidation rate and the different transport mechanisms in the case of the activator treatment lead to the formation of nanoporous Nb and Mo-enriched structures which cannot be found in the reference (activator-free) case. 

As could be seen in the EDS results, the dealloying of the TNM-B1 specimen did not lead to a complete elimination of the aluminum and titanium components in the substrate. The final composition of the substrate correlates with the τ-Phase (Ti_4_NbAl_3_) in the Al–Nb–Ti phase diagram. Generally, for all EDS measurements, both the very rough surface topology and the specimen geometry in the case of the powders (curved and small) limit the accuracy of the results, so these have to be considered to be semi-quantitative. 

### 4.2. Ti-Nb Alloy

The homogeneous structure of the untreated particle complies with the well-known β-stabilizing influence of niobium in titanium alloys and with the generally sluggish kinetics of the β-α transformation which, therefore, can be suppressed completely in processes with high cooling rates such as melt atomization [38]. The observed contrast differences between dendrite cores and borders indicate enrichment of the lighter element Ti near the border, which is in accordance with the expected solidification behavior and also with the results presented by [39]. 

The findings from the activator-treated TiNb42 particles follow, relatively closely, those from the TNM-B1 alloy. The surfaces of the activator-treated powder particles are covered by a thick oxide scale. Beneath the scale, a porous structure is found. The outermost layer of the oxide scale is, as for TNM-B1, enriched in Ti. The main difference in contrast to the TNM-B1 powder is that, for TiNb particles, the porous region has a double-layered structure, and the porosity continues throughout the whole particle core. The comparison of the dendritic structure of the original powder with the pore structure in the core of the activator-treated powder suggests that the Ti-enriched dendrite border areas are preferably dissolved. This can be explained on the one hand by the higher oxygen affinity of Ti; on the other hand by the more restricted reaction of Nb with Cl. The energy of the formation of Nb-chloride is relatively low [32]. This is illustrated by the fact that in the literature, Nb can be found as a substrate for pack cementation processes but not as active deposition material [40]. Oxygen contents, determined by means of EDS, must commonly be considered with great reservation. In the present measurements, the O contents in the various layers of the treated TiNb particle are relatively high, even in the particle core. The highest content is found in the outer scale layer. Nb and Ti can form several binary oxide phases (NbO, NbO_2_, Nb_2_O_5_, TiO, TiO_2_, Ti_2_O_3_, Ti_4_O_4_, or Ti_3_O_5_) but the ternary phase TiNb_2_O_7_ is also known. At a temperature of 1100 K, which is close to the temperature of the activator treatment, the energies of formation of the low-oxygen phases TiO and NbO are lower than those of the other oxide phases [41,42]. Therefore, especially for the outer scale layer, one should expect higher oxygen contents than were actually measured. On the whole, the situation regarding oxygen is still not well understood and needs further investigation. 

### 4.3. Cu-Al Alloy

The change in the particle color is not an unambiguous indicator of a dealloying effect as it can be caused both by Cu enrichment and by the formation of the oxide AlCuO_2_. However, the results of the SEM investigations indicate that aluminum was indeed transferred from the precursor Al-Cu alloy to the Y-zeolite. Interestingly, the transfer occurred at temperatures well below the typical process temperatures of pack cementation. According to Lang et al., who investigated the modification of dealuminated Y-zeolite by reaction with AlCl_3_, the Si-Al changed from 116 to 20; accordingly, the Al content of the zeolite increased from 0.138 mmol/g to 0.753 mmol/g [24]. Lang suggested two mechanisms for the post-synthetic insertion of aluminum atoms into the zeolite framework: (a) insertion into silanol nests and (b) a reaction of AlCl_3_ with the intact SiO_2_ framework (see below). The Si:Al ratio of the Y-zeolite used in these experiments was 2.7, which is considerably lower than in the present case. The lower aluminum uptake capacity associated with it, as well as the increased oxygen content of the treated specimen, suggest a combined dealloying: direct aluminum uptake by the zeolite and binding of aluminum in oxide form, similar to the experiments performed on the TiAl alloy. Indeed, the EDS measurements showed that the smooth regions of the powder surface, as well as an underlying layer, are rich in oxygen and have a higher Al than Cu content. The most probable oxygen source is water adsorbed in the Y-zeolite framework (residual water content was appr. 3 wt%). Furthermore, small oxygen traces in the atmosphere cannot be excluded (the furnace was equipped with high-vacuum, not ultra-high-vacuum). As the energy of formation of Cu oxide is much lower in comparison to silicon oxide, a transfer of oxygen from the zeolite framework is not expected.

Apart from the transport of Al via volatile halogen compounds, the transport of other components, e.g., Cu from the precursor and Si from the zeolite, could also be envisaged. This of course would influence any structure formation in the precursor substrate. However, the fact that copper substrates are diffusion-coated with Al and Mg by means of pack cementation indicates that the mobility of Cu under pack cementation conditions should be very low [17,43]. In comparison to volatile Cu compounds, the formation of volatile Si compounds is more probable. According to Lang, the formation of SiCl_4_ through interactions between AlCl_3_ and the zeolite is possible according to Reaction [24]:

(SiO_2_)_x_(s) + 4AlCl_3_(g) → Al^3+^[(AlO_2_)_3_(SiO_2_)_x−3_](s) + 3SiCl_4_(g). (3)

However, in the EDS measurements of the activator-treated precursor alloy, no indication of Si enrichment was observed. Sodium, as a further candidate for an exchange reaction according to Equation (2), was also not determined by the EDS measurements.

## 5. Conclusions

The starting point of the current investigation was the question of whether component transport via volatile halogen compounds can be used for the dealloying of precursor alloys and employed for the purposeful generation of porous metal structures on a sub-micron scale. The advantage of such an approach would be the processability of metals that are not suitable, e.g., for dealloying in aqueous solutions, and of precursors in a large variety of shapes. Though certain aspects of such component-selective reactions can be found in the existing literature, to the knowledge of the authors, this approach has not been followed in view of producing porous metals so far. Feasibility studies covering different precursor alloys were carried out, including Ti-43.5Al-4Nb-1Mo-0.1B (TNM-B1), TiNb42, and AlCu. Both dealloying effects and micro-scale pore formation were observed. The detailed size of the porous structures observed was in the range of 50 nm for both TNM-B1 and TiNB42, and 500 nm in the case of AlCu. 

The investigated dealloying approach is based on chemical transfer in the gaseous atmosphere of the reaction container. This offers extended processing options. However, the underlying chemistry is rather complex (including, e.g., a large number of competing volatile compounds) and still not fully understood. The formation of NP structures is controlled by the interplay of dealloying (removal of specific elements) and solid-state diffusion (repositioning of the remaining elements). To generate sufficiently high dealloying rates, higher process temperatures will be needed depending on the respective (e.g., Al- or Ti-) halogen compounds. This restricts the use of low-melting precursor alloys as these will experience increased coarsening phenomena at elevated temperatures. 

Generally, the novel approach presented here can be envisaged to be applied to a large variety of materials. Nevertheless, further investigations will be needed to exploit its full potential as a candidate for future NP metal production. 

## Figures and Tables

**Table 1:** Results of EDS measurement as indicated in Figure 12.

Layer		Composition in at%			Ratio
		Ti	Nb	(O)	Nb:Ti
1	Outer scale layer	55	4	41	0.07
2	Dense intermediate layer	26	54	29	2.1
3	Porous layer	10	56	34	5.6
4	Core	34	31	35	0.9
	Precursor (ref., nominal)	58	42	Not def.	0.7

## Data Availability

Data are contained within the article.
